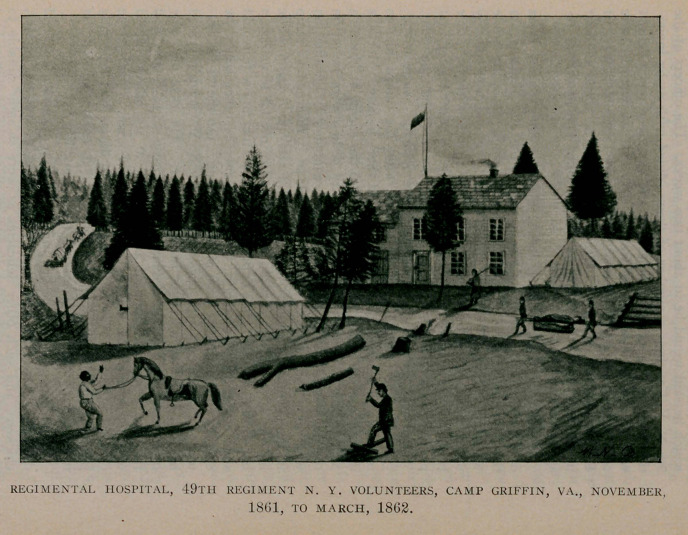# Three Years with the Army of the Potomac—A Personal Military History

**Published:** 1911-08

**Authors:** William Warren Potter

**Affiliations:** Buffalo, N. Y., Brevet Lieutenant Colonel, U. S. Volunteers; Surgeon in Charge of First Division Field Hospital Second Army Corps; Surgeon 57th Regiment New York Volunteers; Assistant Surgeon 49th Regiment New York Volunteers; Recorder Second Division Hospital Sixth Army Corps, etc., etc.


### Three Years with the Army of the Potomac—A Personal Military History

By William Warren Potter, M.D., Buffalo, N. Y., Brevet Lieutenant Colonel, U. S. Volunteers; Surgeon in Charge of First Division Field Hospital Second Army Corps; Surgeon 57th Regiment New York Volunteers; Assistant Surgeon 49th Regiment New York Volunteers; Recorder Second Division Hospital Sixth Army Corps, etc., etc.

### The Grand Review.

On November 20 General McClellan reviewed the larger part of the army of the Potomac at Bailey's Cross Roads, where there was a suitable field, so centrally situated as to be within easy reach of both the right and left wings of the army. The troops participating comprised seventy regiments of infantry, thirty batteries of artillery, and ten or twelve thousand cavalry, in all about sixty thousand men. The President, Secretary of War, and other distinguished public men were present and participated officially in the ceremony. Besides these there were seven Major Generals and twenty-one Brigadiers, with their large and imposing staffs, together with a superb cavalry escort and innumerable bands of music, all contributing to the grandeur and magnificence of the occasion; and when to these are added the thousands of civilians, many of whom were ladies, who crowded the available space as spectators, it will be readily understood what an interesting picture the scene presented to the eye. The roar of cannon, too, added not a little to its imposing splendor. Finally, when all was ready, the trumpets pealed forth the signal for the start, and this mass of living humanity moved forward at the beck of one man, constituting a pageant of magnificent splendor. This was the largest review of troops ever held on this continent up to this time. The 49th did not participate, but was left in charge of the camps of the division, and to support the pickets if necessary. I went, however, and secured a position opposite the reviewing officers, so I had a good opportunity to see them all. This was the first time I had ever seen President Lincoln; he rode in review in a tall hat, and seemed to take kindly to the self-imposed task, though I fancied he was glad when it was over. Two days afterwards General Smith again reviewed our division, which again ended in a sham battle. The General's wife, a beautiful young woman, rode gracefully at his side, entering with great zest into all the details of the parade.

Dr. Wilcox, Captain Washburn, Lieutenant Adams, and other officers of the 21st visited us on the 24th, and we entertained them at dinner, serving them to roast beef, roast lamb, and the like. They seemed highly pleased with our hospitality, and invited us to visit them in return, that they might reciprocate.

Colonel Alberger and I dined in Washington on Thanksgiving Day, November 28, at Mr. Wells's of Fifth Street. This family were friends of the Colonel, and I was invited as his friend. The 9th New York Cavalry, from Chautauqua County, arrived in Washington Thanksgiving night, and I visited the regiment at Soldier's Rest next morning. I had many friends and acquaintances in that regiment, among them E. H. Wilder, and H. G. Parker, of Varysburg. I escorted some of them through the Capitol and other places of interest.

The 49th had two men wounded on picket December 2, which was our first taste of blood from the enemy. On the 5th, Alderman A. A. Howard, of Buffalo, visited us and spent some time with me. His visits had relations to the settlement of the accounts growing out of the expenses of raising the regiment, and we had to supply the necessary vouchers. About this time we became pretty well convinced that we would remain in Camp Griffin during the winter, and some of us, therefore, commenced to consider the propriety of fetching our wives after the holidays, to visit us for a few weeks. I wrote to mine to make herself ready to come, and that I should send for her by Mr. Harry Harbeck, our sutler, who was a very genial gentleman, an agreeable companion, and the personal friend of many of us. He intended to start about the 15th, and return near the 1st of January.

Our first death in camp occurred on December 7th. One of the men of Company B, who had suffered with measles, died from the secondary effects thereof. On this day the Honorable Burt Van Horn and wife, of Niagara County, visited us and dined at our mess. On December 6th, the whole division, consisting of thirteen regiments of infantry, two batteries and one cavalry regiment, went out near Vienna foraging, and brought in two hundred wagon loads of different kinds of forage, starting at daylight and returning at seven o'clock P. M.

Our Regimental Hospital had been established at a deserted house, half a mile in our rear on the road to Chain Bridge, since the first of the month, previous to which time it had been a part of the Regimental Camp. On Sunday, December 8, General Brannan inspected the hospital in its new quarters, approved of the general plan, but directed that the assistant surgeon be required to reside with it; accordingly I took up my abode there on the 9th. I have an India ink sketch of this hospital, made at the time by one of the soldiers—I don't recall his name—which is an excellent picture.

On the 10th Colonel Alberger received a telegram from his wife, who was one of the first ladies to move towards paying us a visit, announcing that she had started for Washington. On the 12th, during one of my incursions to Washington, I visited both houses of congress in session; and, as this was the first view I had taken of the national legislature at work, the scene impressed me greatly. As I became more familiar with congress in after years, this early impression of the members and the grandeur of their work was removed, or very much modified. During these days the weather was most charming by day, and the nights even so mild that I did not need a fire. The bands of the several regiments gave evening concerts, filling the air with lively strains of patriotic and other martial tunes, which served to enliven the otherwise dull life that the officers led in the evening. While we were busy in the daytime enough, the evenings, as a rule, wore heavily on our hands at this time.

The good ladies of Cowlesville, N. Y., through Mrs. Bruce Millar and Mrs. Gilman, sent our regiment a very nice box of hospital stores that we received on the 13th. The goods and fruits were turned over to me, and I distributed them among the sick. The officers met, passed appropriate resolutions, and forwarded them to the ladies. I was, of course, one of the members of the committee to do this.

REGIMENTAL HOSPITAL, 49TH REGIMENT N. Y. VOLUNTEERS, CAMP GRIFFIN, VA., NOVEMBER, 1861, TO MARCH, 1862.

Mr. Harbeck left for the North on the 15th and was instructed to fetch Mrs. Bidwell, Mrs. Tillinghast and Mrs. Potter back with himself and Mrs. Harbeck when he should return, which he said would be in about two weeks. The officers of the 9th N. Y. Cavalry—a number of them—dined with us by invitation the day of Harbeck's departure, and expressed much delight with their entertainment, as well as envy that we were to have our wives with us. Mayor Alberger and Isaac Holloway, of Buffalo, visited us on the 17th and the Mayor spent an hour in my tent, where a number of officers called and paid their respects to him.

Rumors of an impending battle filled the camp during these days; and, finally, as if to give verity to the coy Madame, on the 20th the long roll was sounded throughout the camp while we were at dinner. The regiment turned out and was speedily under arms, when orders came for it to march with the other troops of the division towards Drainesville. I started after first paying the sick a visit, which delayed me about half an hour. When near Lewinsville I overtook the Brigade Surgeon, Dr. Herrick, who ordered me back to look after the camps, and to be ready to receive the wounded, should there be any. McCall's division, on our right, had a skirmish with the enemy at Hunter's Mill, killing a number—rumor said sixty bodies were found on the field—and losing a few killed and wounded. Our division went as a support, but was not needed, so it returned to camp in the evening. Colonel Alberger's wife arrived in camp while he was away on this expedition, so it fell to my lot to entertain her until his return. She expressed the hope that my wife would soon come, that she might, by the presence of other ladies, be enabled to spend more time in camp herself. On the 21st, Lieutenant Carson, of North Java, and the 9th N. Y. Cavalry, visited me and remained all night in my quarters. He gave me some interesting news of Dr. Adams, of the gossip at his home, and the like. General Brannan, and his staff of six officers, dined with us on the 21st also, by invitation of Colonel Alberger, the General escorting Mrs. A. to the table. Our dinner was especially prepared for the occasion, and all seemed to enjoy the event, as it lent a little variety to the social part of our life.

On Christmas Day the sick in hospital were furnished a special dinner by Dr. Hall and myself, consisting of oysters, turkies, chickens, and such other delicacies as were thought suitable to the occasion, and to their stomachs. From this time until the end of the year I was busy, during all spare time, with the quarterly return of medical and hospital property, and stores. The return was a complicated and a perplexing one to make up at first; but

I finally mastered it, finishing it on the 31st of December, the last day in the afternoon. That evening my wife arrived, and, as Colonel Bidwell had gone to Washington to meet his own wife, we occupied his quarters that night and during his absence.

1862.

New Year's Day, 1862, was a warm, delightful day—so much so that we spent nearly the whole of it out of doors, without either overcoats or wraps. It was dusty, too, and reminded one of early October at the North. We had a special dinner in observance of the custom as in all well-regulated households; and the day was generally observed as a holiday throughout the army, only the necessary duty being required of the troops.

During the first two or three weeks of my wife's visit we were quartered at the hospital, as I had been before her arrival; but after the middle of January the rains were so constant, that I moved back to the camp again, to avoid walking back and forth to meals in the mud, Dr. Hall taking my place at the hospital. The presence of so many ladies in camp made it quite interesting to the officers and soldiers, who seemed to enjoy this little infusion of home life into ordinary military routine. The ladies, themselves, enjoyed it greatly; the troubles and inconveniences resultant from such restricted quarters they made light of; while they readily took advantage of everything which could be made to contribute to the general good or amusement, no matter how slight or trifling in character it might ordinarily have seemed to be. Evenings were spent in visiting each other, generally one of them inviting all the others, where conversation, singing, and eating, with some card playing served to while away many a delightful hour; and in this way the months of January and February slipped away, all too rapidly.

The latter part of January brought Dr. McCray to visit us, and we enjoyed the week of his sojourn very much indeed. He, likewise, fell into our easy-going ways with alacrity, and departed with regret. We were really a very jolly lot, skimming the cream of the hours as they flew by, and I now look back upon this winter at Camp Griffin as among the pleasantest of my life. We occasionally visited Washington staying for a night and a day; but not often, for we one and all, preferred the novelty of our camp life to the attractions of the capital.

Thus affairs went on day by day until the Ides of March; then came certain premonitions of a probable early move in the way of preparatory orders, but nothing definite until, suddenly, in the middle of the night of Sunday, March 9, an orderly galloped into camp, his horse's hoofs ringing crisp over the hardened ground, with orders to get ready to move immediately. A

little later in the campaign such an order, even in the dead of night, would not have disconcerted us in the least; but now, with all our comforts around us, and our wives still with us, we were startled as out of a dream, and everybody flew around without method or purpose, doing the most unnecessary things, and saying the most disagreeable things imaginable. However, the regiment was aroused, breakfast cooked, and we were under arms at daylight, in waiting for the final order which should send us forth to do and to save. The ladies were left in care of Mr. Harbeck, our good and trusted coadjutor and friend, who took them all to Washington during the day (Monday), while the division marched out as far as Flint's Hill, near Fairfax C. H., and bivouacked for the night, our first day's march covering about twelve miles through the mud. And so we bade this hasty and unceremonious adieu to Camp Griffin, the dear old spot, where we had had so many pleasant experiences, and where we had gone through so much of that tutelage so necessary to make us efficient soldiers.

Our first night at Flint's Hill was a pretty severe trial to me, at all events. I slept in a D'Abri tent for the first time, pitched on a side hill, and but just large enough to permit me to lie down in. I did not take off my clothing that night, even sleeping ( ?) in my overcoat, hat and spurs. The night was windy and trying, and of course I got but little sleep, fearing all the time we might be roused by orders to move. Finally morning came, finding us all quiet, and we remained at Flint's Hill until Saturday, the 15th of March. On Thursday, March 13th, the Rev. Dr. Sunderland of Washington, and the Rev. Dr. Heacock of Buffalo visited us, the latter remaining all night with his brother, Captain R. B. Heacock, who was killed at Spottsylvania C. H., May 18, 1864. Thursday night we received orders to be ready to move at a moment's notice, and Saturday morning, the 15th, we marched to Fairfax C. H.,thence towards Alexandria, camping in the vicinity of the latter at four o'clock P. M. It rained hard all day long and at night we were a sorry lot; but a big camp fire, around which the field and staff pitched their tents, served to cheer and dry us. It also afforded us an opportunity to cook a hot supper, and I enjoyed broiling the fresh beef on a forked stick, which tasted sweeter than though it had been served from Delmonico's grill.

The next day, Sunday, the 16th, I received information that my wife was in Alexandria, whereupon I immediately obtained a pass and visited her at the City Hotel. It appears the ladies remained in Washington for a few days to learn, if possible, what our destination would be, and, upon learning that we were ordered back to the vicinity of Alexandria, they took the boat over from Washington Saturday evening, sending us word to that effect Sunday morning. The party consisted of Mrs. Tillinghast, Mrs. Bidwell, Mrs. Alberger and Mrs. Potter, and their husbands visited them about noon of Sunday at the City Hotel, as before stated. We remained there until Monday, when I bade my wife good-bye, not to see her again until the last day of the year, December 31, 1862, when I went north for a few days upon my promotion to Surgeon of the 57th Regiment N. Y. Volunteers.

Our command remained near Alexandria for a week, awaiting transportation to Fort Monroe; for McClellan had by this time determined to adopt the Peninsula route to Richmond. On Sunday P. M., March 23, we embarked at Alexandria on the "Arrowsmith," a Long Island Sound steamer chartered for the purpose. We anchored for the night off Mount Vernon, as it was even then not safe to sail down the river at night, on account of the possibility of encountering rebel batteries, setting sail next morning for Fort Monroe, where we arrived the same evening, but did not debark till Tuesday morning. General Davidson and staff came with the 49th on the Arrowsmith. He was our Brigade Commander now, having in January succeeded General Brannan, who had been ordered to the South Carolina coast, taking with him the 47th N. Y. The 77th, Colonel McKean, was assigned to us in place of the 47th, making our brigade consist of the 33d, the 49th, and 77th N. Y., and the 7th Maine volunteers. I took supper Monday evening and breakfast Tuesday morning at the Hygeia Hotel with Mr. Harbeck, and during the forenoon we took up our march towards Newport News, where we encamped for the night. We halted for dinner at Hampton, which had been burnt the summer previously by General Magruder. Hampton had been a pretty village, built almost entirely of brick, and had a population of about 2,500 inhabitants before the conflagration. It was frequented as a summer resort before the war, by wealthy Southerners, and was a place of much aristocratic fame.

Before leaving Old Point I paid a visit to Fort Monroe, where I saw the then celebrated "Union" and "Floyd" guns. These were the two heaviest guns we had ever cast, and were attracting much attention in ordnance circles just then. The Fort impressed me with its ponderous magnificence, enclosing as it did and does between thirty and forty acres of ground, and mounting the heaviest ordnance we were capable of producing.

Colonel Bidwell was left at Washington sick when we sailed and to Lieutenant Colonel Alberger devolved the command of the regiment. Dr. Hall was also left behind to look after the sick and the property of the brigade, to report in person as soon as that duty was performed. This left me in medical charge of the regiment, and the first surgery I had was the extraction of a ball from the wrist of one of the men on March 25, fired by some soldier in a neighboring regiment.

The officers were now without tents, excepting the D'Abri or shelter tent; but I used a hospital tent for my quarters, which the Major and Quartermaster occupied with me. At Fort Monroe I had a glimpse of the "Monitor," which had then so lately fought the most celebrated naval battle of the age within sight of our present camp, where the spars of the "Cumberland" were yet standing obliquely out of the water, a monument to the death of wooden vessels of war.

March 27th. Smith's Division made a five mile reconnoisance in the direction of Yorktown, driving some cavalry videttes away in the vicinity of Young's Mills. We remained out all night, bivouacking on lines lately occupied by the enemy, and returned to Newport News next morning, camping on the banks of the James about a mile above the latter point. Our camp was named "W. F. Smith," in honor of our Division Commander, who was familiarly known as "Baldy," a soubriquet he acquired at West Point when a cadet. During the few days we were here we frequently obtained fresh oysters from the Bay, and by March 30th, I noticed peach trees in blossom. I picked up a porcupine-fish skeleton on the beach at Newport News on the 31st that I sent, home and still have in my possession. (Now in the Buffalo Historical Society Building.)

We now began to see the desolation of war—all houses were in ruins—and scarcely a living thing, "native and to the manner born," remains. The inhabitants, excepting the slaves, fled with retiring foe, leaving not even a stray chicken for the dreadful "Yankees." Newport News is not a town, as might be supposed, but simply a military post where 5,000 men are quartered in barracks, under the command of General Mansfield. On March 31st the rebel gunboat "Teaser" steamed down the James, and commenced throwing shells at our camp; but, after discharging three shots, she turned around and went back to Richmond, having done no damage.

April 1st. From this time I shall write generally in the present tense, as it is the most convenient. Today we made another reconnoissance in the same direction as before. We deployed a battery and fired a few shots at some cavalry, returning to camp about three o'clock P. M., without accident or casualty. The field and staff being still without tents, I borrowed a hospital tent of Dr. Mulford, surgeon of the 33d, and we are all occupying it as one family. Colonel Alberger is not well but keeps about camp, though Major Johnson is in command. The comforts of

Camp Griffin are often referred to these days, in contrast to the beggary of our present life.

Friday, April 4th. At six o'clock A. M. the army took up its advance upon Yorktown, our division leading on this line. We marched ten miles the first day, reaching and bivouacking in the vicinity of Young's Mills. Here we find barracks for three regiments, which were so lately deserted by the enemy that fires are still smouldering in the chimneys. We started again this morning, April 5th, at daylight, our brigade leading the march of the 4th corps. We found the enemy before noon, who resisted our advance in the rain, and we spent the P. M. in skirmishing, losing, in the 49th, one killed and one wounded. By four o'clock we had reached the position that the enemy sustained along the Warwick River, the strength of which proved sufficient to bring us to a halt. We established our pickets within three or four hundred yards of the enemy's works, and lay on our arms during the night. We were in a piece of woods in which it was difficult to maneuvre artillery, where we lay during Sunday and Monday, the 6th and 7th. Shell from the enemy's batteries frequently fell amongst us, and on Sunday the 49th had five men wounded. I picked up a spent cannister shot, that only expended its momentum at my feet. It was now evidently determined to siege the enemy's position as, on Monday evening, we were withdrawn to a safer and more comfortable position, after having been about sixty hours under fire. This withdrawal was accomplished with difficulty, the utmost secrecy being maintained, lest the enemy should become aware of our purpose. I remember an incident that will illustrate the caution observed: in falling back two of my hospital attendants were carrying some camp kettles on a pole, when the rattling of the kettles attracted the attention of General Davidson, who was sitting on his horse all alone in the woods where we were passing. He flew into a great rage, scolding me soundly for permitting the men to make such a noise. It was nearly dark and raining at the time, and I reached our appointed place in anything but good humor.

(Continued.)

## Figures and Tables

**Figure f1:**